# Acknowledgment to the Reviewers of *Journal of Cardiovascular Development and Disease* in 2022

**DOI:** 10.3390/jcdd10020033

**Published:** 2023-01-17

**Authors:** Journal of Cardiovascular Development and Disease Editorial Office

**Affiliations:** MDPI AG, St. Alban-Anlage 66, 4052 Basel, Switzerland

High-quality academic publishing is built on rigorous peer review. *Journal of Cardiovascular Development and Disease* was able to uphold its high standards for published papers due to the outstanding efforts of our reviewers. Thanks to the efforts of our reviewers in 2022, the median time to first decision was 20 days and the median time to publication was 39 days. Regardless of whether the articles they examined were ultimately published, the editors would like to express their appreciation and thank the following reviewers for the time and dedication that they have shown *Journal of Cardiovascular Development and Disease*:
Alberto Francesco CeredaKyoung-Min ParkAleksandar JovanovićLidija PoposkaAleksandra ŻebrowskaLingwei WangAleš BlincLõpez-Candales AngelAlessandro BarboneLoredan NiculescuAlessandro BiffiLouis Maximilian BujaAlessandro MalobertiLu WangAlessandro PiniLuca FontanaAlessandro ZorziLuca MarinoAletta MillenLuca Salvatore De SantoAlex Cleber Improta-CariaLucia MirabellaAlexander B. A. VonkLucia StančiakováAlexander BerezinLucile HouyelAlexander E. BerezinLucile MiquerolAlexander OberhuberLudmila Danilowicz SzymanowiczAlexandru BejinariuLudwig MuellerAlexandru BurlacuLuigi CappannoliAlexey SuminLuigi GianturcoAlfredo CaturanoLuigi LovatoAlfredo CesarioLuisa CacciavillaniAlice BonomiLukasz MalekAlicia Rodríguez-BarberoLynn A. FussnerAlireza GheiniLyuba Dineva MitevaÁlvaro García-OsunaMaciej SińskiAna Cristina Silva RebeloMaciej StaporAna González-SuárezMagdalena Markowicz-PiaseckaAna Isabel PadrãoMahmoud DiabAna VujicMakoto FurugenAnand Prakash SinghMakoto OnoAnders Erik Astrup DahmMakoto SugiharaAndrea AlexandreMalene JørgensenAndrea BaragettiMan LiuAndrea BuonoManal Mohammad AbbasAndrea GrilloManel Sabaté TenasAndrea M. AlexandreMarc DorenkampAndrea MontaltoMarcello IasielloAndrea SzékelyMarcin BasiakAndreas M. BeyerMarco Alfonso PerroneAndreas PflaumerMarco FoganteAndreea Catarina PopescuMarco GuglielmoAndrei VasilateanuMarco Matteo CicconeAndrew AtkinsonMarco MongilloAndrey LozhkinMarco SchiavoneAndrzej CiechanowiczMarco ZuinAndrzej GlowniakMarcus BrooksAndrzej KułachMarcus RedaélliAndrzej PrzybylskiMarek KiliszekAneta AleksovaMargarita A. SazonovaAngela PucciMaria AndreassiAnil Kumar JonnalagaddaMaría Angélica RivarolaAnn C. FoleyMaria Giovanna LupoAnna E. PłatekMaria IasconeAnna Kablak-ZiembickaMaria Luisa ZeddeAnna LisowskaMaria MirabelliAnna Olasińska-WiśniewskaMarian SimkaAnna StarshinovaMariana FloriaAnna VaskuMariann HarangiAnna Vittoria MattioliMarieke StammesAnnunziata NuscaMarika MassaroAnton KiselevMarisa VarrentiAnton KutikhinMark M. GallagherAntonia MancusoMarko LaaksonenAntonin TrimailleMarkus BergerAntonio BarileMarkus PuchingerAntonio NennaMarta Nowakowska-KotasAntonio RizzaMartin BesserAntonis SakellariosMartin HartrumpfArend Derk Jan Ten HarkelMartin Keith JohnsonArmando CaseiroMartin MartinekArun SamiduraiMartina BrianiAshfaq AhmadMartina PeršeAshley McPeekMarzenna ZielinskaAtiyeh AbdallahMarziliano NicolaAtsuhiko YagishitaMasaki IzumoAttila FrigyMasashi YamashitaAttila OláhMasatake KobayashiAttila RokaMassimo CapocciaAttila ThuryMassimo IacovielloAttilio RestivoMateusz SpiewakAvraham ShotanMathieu JozwiakAxel SchlittMatteo BertiniAziz MominMatthew BrodyAzizah UgusmanMatthew GrantBarbara BauceMatthew J. WolfBarbara Kutryb-Zaja̧cMatthew W. MartinezBart De GeestMatthias HermannBéatrice Brembilla-PerrotMatthias M. LoebeBenincasa GiudittaMattia ChiesaBerenice Caneiro-QueijaMaurizio Cappelli BigazziBernhard RauchMauro MalvèBernhard ReimersMazhar Javed AwanBert-Ram SahMd. Abul Hassan SameeBibhuti B. DasMenachem NahirBin ZhouMicah ZuhlBirte WeberMichael D. SpartalisBlake CochranMichael LichtenauerBortolo MartiniMichael RubartBradley KellerMichael TsangBrian D. LoMichail E. KlontzasBruno BrandoMichal KidawaBruno José Nievas SorianoMichal LewandowskiBruno PassarettiMichel PucéatBruno TrimarcoMichelangelo LucianiCalin CorciovaMichele CostanzoCamden HebsonMichele GolinoCamilla CalvieriMichelle TallquistCarl J. LavieMichinari HiedaCarlo CampanaMie KurataCarlo PalomboMiguel Ángel Castro VillamorCarlos López-de-CelisMiguel LozanoCarmen C. SucharovMiguel Rebollo-HernanzCarmen SpaccarotellaMikael DellborgCarmine PizziMilan PrsaCatalina LucaMilka KoupenovaCaterina BonfiglioMilorad TesicCatherine N. BagotMin Tsun LiaoCedric ManlhiotMin XieCees A. SwenneMingming TongChao LiMing-Sheng TengChao ZhouMiodrag JanicCharles PorterMiran ŠebeštjenChi Keong ChingMiroslav BarancikChia-Hung ChouMiroslava KvandováChiara MacchiMiroslawa PuskulluogluChih-Cheng WuMislav VrsalovicChih-Hsin HsuMitsuru OhishiChih-Ming LinMohammad Arif Sobhan BhuiyanChing-Hui SiaMohammad S. ImtiazChristel WeissMona M. El RefaeyChristian BrinkmannMonika KoziełChristian GrebmerMoreno ZanardoChristian JuxMoritake IguchiChristian MeyerMoshe Rav AchaChristine TørrisMossab Y. SaeedChristof VulstekeNai-Jen ChangChul Ho KimNaim MahroumChun Woo ChoiNarayanaswamy VenketasubramanianClarence KhooNarcis TribulovaClaude FranceschiNashwa El-KhazragyClaudia CrociniNatalia Reglero-RealClaudio De LuciaNataša Marčun VardaClaudio HumeresNatasja M.S. De GrootClinton WebbNicholas GrubicConcetta Di NoraNicholas KounisCorey StiverNiclas BjarnegardCrina Julieta SinescuNicola FerraraCristian MornoşNicola MaureaCristian StătescuNicolò SistiCristina AurigemmaNikolaos FragakisCristina BallaNikolaos KaramichalakisCristina CiucaNing LiCristina P. VicenteNir NesherCristina RodríguezNobuyuki IshibashiCynthia YeungNobuyuki OhteDagmar PavlůNyeonju (NJ) KangDamian HudziakOlga AfanasievaDamir FabijanićOmer DzemaliDana PopOscar CampuzanoDaniel Bia SantanaOsman O. Al-RadiDaniel BiermannPan GaoDaniel GoldmanPantelis ZempekakisDaniel LighezanPaola GargiuloDaniel WendtPaola PiscopoDaniela MastroiacovoPaolo AngeliniDaniele LinardiPaolo BerrettaDaniele MasaronePaolo SeverinoDavid DolivoPaolo VersacciDavid SchumacherParamjit S. TappiaDavid SedmeraParwis MassoudyDavid ZweikerPascal J. LafontantDeborah HendersonPascale GaussemDemetrios ArvanitisPaško KonjevodaDerek L. TranPasquale MastrorobertoDesiree Nadine WusslerPasqualino SirignanoDhaval ChauhanPatnana Syamasundar RaoDiana ŽaliaduonytėPatrizio MazzoneDimitrios PatouliasPaul GrossfeldDmitri ShchekochikhinPaulina KoczurkiewiczDomen PlutPaulo GentilDominga IacobazziPaulo Magno Martins DouradoDominik S. SkibaPavel JansaDonato D’AgostinoPavel Vladimirovich DolganovDrew NassalPavel ŽáčekEdit HermeszPaweł GaćEduardas SubkovasPawel KleczynskiEduardo M. VilelaPaweł KrzesińskiEduardo SternickPaweł WańkowiczEdward James Frazer DansonPeter A. KavsakEdyta Ewa SutkowskaPeter PaalEfrat NeterPeter R. KoenigEhud ChorinPetr NachtigalEiichi WatanabePetros IoannouEitaro KodaniPeyman RezaieEleftherios KaratzanosPhilipp S. LangeElena GalliPhilippe J. Van RosendaelElena ReffoPiero PaladiniEleonora VatamanPierpaolo PalumboElif Inan-ErogluPierre Yves R. MarcyElisa CairrãoPierre-Simon JoukElżbieta Poniedziałek-CzajkowskaPiotr DuchnowskiEmad Al-DujailiPiotr FutymaEmanuele BertagliaPiotr MichelEmiliano Raúl DiezPolyxeni MantzouratouEmilio GrecoPraveen Kerala VarmaEmma C. CacciolaPrithwish BanerjeeEmmanuel BourdonPriya ChockalingamEndre NemethQiangjun CaiEnkhmaa ByambaaQin Xiang NgEnoch Odame AntoRada EllegårdEnrico FerrariRade M. BabićEnrique Berjano-ZanónRadosław GocołEraldo OcchettaRafał JanuszekErik BehringerRafał MańczakErik FungRaffaele SerraErnesto JimenezRaffaella MottaEstefanía OliverosRaimundas RažanskasEugen SandicaRajani GeorgeEugenio MartelliRamachandra BarikEvangelia KouidiRamón González-CamarenaEvgeny BelyavskiyRana O. AfifiEwa MichalakRashad ZayatEwa Straburzyńska-MigajRasmus RiviniusFabio MiraldiRatko LasicaFabrice BauerRaymond C. C. WongFabrizio MonacoRebecca TsaiFaisal Fiazuddin SyedRegina Steringer-MascherbauerFatih YalcinReuben HowdenFatimah KhalafReza ArdehaliFatme AlAnoutiReza MortazaviFederica FogacciReza Shekarriz-FoumaniFei LiRiccardo BarianiFelice SiricoRich GoodwinFelix B. EngelRichard H. Parrish IIFeng GaoRichard TrohmanFeng ZhuRobert KrysiakFerdinando IellamoRobert ZymlinskiFernanda Cristina Paccola MesquitaRoberto De PontiFevzi YılmazRodolfo BuselliFilippo MiglioriniRoger HullinFilippos TriposkiadisRolf BodmerFlavia FranconiRosa SuadesFlavio TarasoutchiRyan D. SullivanFlorian WeinbergerRyota KobayashiFrancesca ZimettiSachiko Miyagawa-TomitaFrancesco BedogniSahrai SaeedFrancesco GiallauriaSakiko MiyazakiFrancesco MassariSalvatore PastaFrancesco NappiSameer HirjiFrancesco PatanèSameh SaidFrancisco Eugenio Calvo-IglesiasSamuel MenahemFrancisco NayaSandeep JainFranck PaganelliSarah BeckFrederick SchoenSaša FrankFukiko IchidaSatoshi ShizutaFumihiro OgawaSean M. WuGabriele GhettiSebastian KelleGaetano ThieneSebastian SchnaubeltGal TsabanŠebeštjen MiranGaryfallia PeperaSemon WuGeorge PorterSergio ContiGeorge SamanidisShah AliGeorge W. VetrovecShailendra SinghGerald H TomkinShaofang NieGermaine Claudette VerwoertShazia JamshedGermaine Cornelissen-GuillaumeSheldon GoldbergGerman Dominguez VíasShelley Shelley MiyamotoGian Luigi NicolosiShengshuai ShanGianLuca ColussiShibu MathewGianluca L. ZingariniShigeki KusaGianluca LuccheseShinya OkazakiGianluca RigatelliShunsuke SaitoGiovanni BottoSi PhamGiovanni CudaSilvia CalabriaGiovanni NanoSilvia LiskovaGiovanni RuvoloSilvia Mas-PeiroGisèle Clofent-SanchezSimcha R. MeiselGiulia FrissoSimon C. EccleshallGiulio Francesco RomitiSimon Christopher BodyGiulio ZucchelliSimon D. BamforthGiuseppe BarlettaSimona ZaamiGiuseppe BorianiSinsia A. GaoGiuseppe CaminitiSławomir KasperczykGiuseppe CilibertiSonia RoncheyGiuseppe GulloSophie JacobGiuseppe MandraffinoSrinivasan VendanthamGiuseppe MasciaStanislav KotlyarovGiuseppe SantarpinoStefan Cristian VesaGizem KeceliStefan LyerGlenn FishmanStefan SchwabGregory P. BartonStefano BallestriGrigorios TsigkasStefano SchenaGrzegorz KopecSteliana GhibuGudrun Maria FeuchtnerStepan HavranekGuido DohmenStephan LindemannGunter KerstStephane CarlierGust H. BardyStéphanie SaxerGustavo Vieira De OliveiraStephen C. Kolwicz Jr.György JermendyStephen GerferHabib RehmanSuet Yen ChongHaekyung Jeon-SlaughterSurendra RajpurohitHaipeng LiuSven DittrichHan KemperSvetlana KlinovaHartmut KuhnSylvie Di FilippoHassan MehboobSzymon BudrejkoHayato TadaTaimur SaleemHeather BloomTakaaki SenbonmatsuHeather C. EtcheversTakao KatoHeiko SternTakashi ObamaHelen PurtillTakeshi AdachiHelmut MairTamas AlexyHeng MaTarek AlsaiedHenry SutantoTatjana PekmezovicHerbert LoellgenTatsuya MorimotoHideki MiyachiTaufiek Konrad RajabHideo WadaTeruhiko ImamuraHilde E. GrootTetsuo SasanoHiroe TobaTetsuya IshikawaHiroki TeragawaTheodor FischleinHirotsugu FukudaTheodoros D. KaramitsosHiroyuki YamagishiThirunavukkarasu SathishHisakazu KatoThiyagarajan VaradharajanHisato YagiThomas FinkHitoshi HachiyaTim A. FischellHolger EltzschigTim PrickettHong MaTina Ulrike CohnertHoraţiu MoldovanTobias RufHorea FeierTomas HolubecHsien-Tsai WuTomasz HirnleHuiling ChenTomasz RakowskiHung-Tsung WuTomasz TokarekIbrahim El-BattrawyTomasz ZdrojewskiIbtihal Al AmriTomasz ZielinskiIgnacio HernandezTommaso BucciIlaria ZanottiTomohiko ShindoIlias P. DoulamisToni LassilaInes MonteTorsten K. MalmInna Rabinovich-NikitinToshihiro TsurudaIoana MozosToshiro KitagawaIoannis K. ToumpoulisTrayambak BasakIoannis ParaskevaidisTullio TesorioIrena IlicTun-Wen PaiIrina Iuliana CostacheUberto BortolottiIsabel RodríguezUgolino LiviIsao ShiraishiUlrich JuliusIstván OsztheimerUtkarsh KohliIstván SzokodiValentina BucciarelliIvan Cruz-AcevesValentina MantegazzaIwona ŚwiątkiewiczValeria ContiIzabella UchmanowiczValeria PergolaJacek BiałkowskiValluvan JeevanandamJacek PiątekVasileiou PanagiotisJacqueline M. BentelVasiliki TsigkouJae Boum YoumVasu D. GootyJae Kyu RyuVedran VelagićJae Min ChoVeera Ganesh YerraJakub PodolecVessela KrastevaJames R. GroomeViacheslav NikolaevJan MalíkVicente ClimentJasmin ArrichVicky WangJason AcworthVictoria GonzalezJason AliVijay KumarJean Jacques MonsuezVijaya Anand ArumugamJeffrey G. JacotVijayakumar SukumaranJelica Grujić-MilanovićVilnis DzerveJere PaavolaVinay Kumar TyagiJesus M. Martin-CamposVincenzo GasbarroJiangbin WuVincenzo NuzziJingli CaoVincenzo QuagliarielloJinli WangVinesh VinayachandranJirko KühnischVinit BaliyanJiuann-huey Ivy LinVivian De WaardJoanna Szczepańska-GierachaVladimir A. ShvartzJoanne Van RynVladimir JakovljevicJoerg-Ingolf SteinVladimiro VidaJohannes SchwabVlasta FesslovaJohn ElefteriadesWei FengJohn PepperWei-Chieh LeeJohn W. BelmontWeilai DongJo-Nan LiaoWenqun LiJonathan J. H. BrayWilhelm MistiaenJong Yun LeeWilliam WangJonica CampoloWinnie ChuaJordan D. AwerbachWojciech DrygasJosé A. BarrabésWojciech JachećJosé AlfieWolfgang BoettcherJosé Antonio Barrabés RiuWouter KokJosé António BeloXi ZhouJose Geraldo MillXianglin L. DuJosé Luis De La PompaXiangyu ZhangJoseph Boone MuhlesteinXian-Wu ChengJoshua M. BockXiaohan FanJuan Carlos GrignolaXiaoliang WangJuan Patricio NogueiraXin LouJuan Ramón GimenoXing ZhangJulia HalbfaßXinli LiJulio GarciaXiongwen ChenJungmyung LeeXueying HuangJürgen ClaesenYalda AfsharJuš KšelaYang PengKalamullah RamliYanming LiKarel AllegaertYasukochi SatoshiKaren OcorrYasushige ShinguKarina Wierzbowska-DrabikYeon Hyeon ChoeKatarina WestlingYin Hua ZhangKatarzyna Mizia-StecYoriyasu SuzukiKatharina ScherschelYoshihiro FukumotoKatherine YutzeyYoshiko KidaKatrin SchäferYu KangKavitha S. RaoYu TaniguchiKazuhiro P. IzawaYu-Cheng HsiehKazuya TateishiYuji KomakiKeiichi IshidaYuji NagatomoKenichi GotoYuji ShimizuKenichiro YamamuraYukihiko MomiyamaKevin L. SackYuliya I. RaginoKevin LachapelleYunfu SunKevin WillyYung Liang WanKhalid Mohamed El-SayYungi KimKichang LeeYuriy I. GrinshteinKlaas F. FranzenZbigniew SiudakKlaus GörlingerZdravka PoljakovičKlaus JungZdravko BabicKlaus-Dieter SchlüterŽeljko ReinerKoichi NaitoZengsheng ChenKonstantin YastrebovZhichao JinKonstantinos A. GatzoulisZhifan GaoKonstantinos PetsiosZhipeng HuKoya OzawaZhonghua SunKozo NakamuraZiad Said DahdouhKristin E. EllisonZiqing LiuKristine M. KrajnakZofia T. BilińskaKrithika RaoZofia Teresa BilińskaKrzysztof J. FilipiakZohra LamiralKrzysztof KaczmarekZoltán VargaKurt BestehornZuzana Višňovcová


